# Direct S-Poly(T) Plus assay in quantification of microRNAs without RNA extraction and its implications in colorectal cancer biomarker studies

**DOI:** 10.1186/s12967-019-2061-6

**Published:** 2019-09-23

**Authors:** Yanqin Niu, Sijian Xia, Mingyang Su, Quanjin Dang, Kang Kang, Li Li, Deming Gou

**Affiliations:** 1 Shenzhen Key Laboratory of Microbial Genetic Engineering, Vascular Disease Research Center, Guangdong Provincial Key Laboratory of Regional Immunity and Diseases, Carson International Cancer Center, College of Life Sciences and Oceanography, Shenzhen University, Nanhai Ave 3688, Shenzhen, 518060 Guangdong China; 2 Key Laboratory of Optoelectronic Devices and Systems of Ministry of Education and Guangdong Province, College of Optoelectronic Engineering, Shenzhen University, Shenzhen, 518060 Guangdong China; 3 Department of Biochemistry and Molecular Biology, School of Basic Medical Sciences, Shenzhen University Health Sciences Center, Shenzhen, 518060 Guangdong China

**Keywords:** Direct S-Poly(T) Plus, Circulating miRNA, Biomarker, Colorectal cancer

## Abstract

**Background:**

Advances in microRNAs (miRNAs) biomarkers have generated disease markers with potential clinical values. However, none of these published results have been applied in clinic until today. The main reason could be the lack of simple but robust miRNA measurements.

**Methods:**

We built up a simple but ultrasensitive RT-qPCR protocol, Direct S-Poly(T) Plus assay, for detecting miRNAs without RNA purification. In this study, the method was optimized and compared with other RNA purification-based miRNA assays, and the sensitivity was tested. Using Direct S-Poly(T) Plus method, seven potential miRNA biomarkers of colorectal cancer were validated.

**Results:**

It is possible to detect approximately 100 miRNAs with minimal plasma inputs (20 μl) and time (~ 140 min) with this approach. The sensitivity of this method was 2.7–343-fold higher than that of the stem-loop method, and comparable with S-Poly(T) plus method. 7 validated miRNA biomarkers of colorectal cancer by Direct S-Poly(T) plus assay could discriminate colorectal cancer stage I from healthy individuals, and promised satisfactory discrimination with the area under receiver operating characteristic (ROC) curve ranging from 0.79 to 0.94 (*p* value < 0.001).

**Conclusions:**

This simple and robust protocol may have strong impact on the development of specific miRNAs as biomarkers in clinic.

## Background

MicroRNAs (miRNAs), a class of single-stranded small RNA with 18–25 nucleotides, are capable of regulating gene expression at the post-transcriptional level by binding the 3′-untranslated region (3′-UTR) of target mRNAs [1–3]. Past researches have proved that miRNAs involve various biological processes, such as cell differentiation, proliferation, and apoptosis [2, 4, 5]. Moreover, dysregulation of miRNAs is implicated in many types of disease, including cancer and cardiovascular diseases [6–8]. miRNAs are not only in cells, but also present in extracellular fluids, including plasma, serum, urine, saliva and milk [9–11]. For example, more than 500 miRNAs had been detected in the serum or plasma [12]. Majority of circulating miRNAs cofractionate with protein complexes and circulate in blood in a highly stable form [13]. More importantly, circulating miRNAs correlate with diagnosis, prognosis and responses to treatment [14–16]. These findings suggest that circulating miRNAs have great potential as biomarkers in monitoring the body’s physiopathology status.

Colorectal cancer (CRC) is the third most common cancer in the world and the second leading cause of cancer-related deaths. It is estimated that over 1.8 million new CRC cases and 881,000 deaths occurred in 2018, which is about 1 in 10 cancer cases and deaths [17]. If diagnosed at an early stage, the mortality of the disease could be potentially reduced. Unfortunately, most patients have no phenotypic symptoms until later stages [18]. Moreover, the current established colorectal cancer screening tests in these years, including colonoscopy, fecal occult-blood testing, and stool DNA test have not been well-accepted, because of their invasive and unpleasant nature, high cost or limited sensitivity [19, 20]. Therefore, more efforts need to be devoted to discovering noninvasive, sensitive and convenient screening test methods, and miRNAs show to be one of the most promising molecular biomarkers for tumor early diagnosis and prediction of prognosis. Up to now, advances in miRNA biomarkers have generated some candidate markers of CRC with potential clinical values [21–23]. However, these published results have not been clinically applicable until today. The possible reasons could be the lack of sensitive and easily applied method in clinic, as well as knowledge about which biomarker(s) are stable and reproducible for clinical use.

Reverse-transcription quantitative real-time PCR (RT-qPCR) method is most frequently used for measuring the expression of miRNAs due to its high sensitivity, specificity and reproducibility [12, 24, 25]. Currently, RT-qPCR is mainly based on RNA purification and small RNA enrichment. During the procedure, it is unavoidable to loss slight RNA during washing and dissolving steps and it may lead to detection failure. Moreover, it is time-consuming and laborious for processing large numbers of samples, which limits clinical application. We had previously developed an assay that could detect the virus from serum specimen without the need for RNA purification [26]. In this study, we optimized an effective RT-qPCR assay for directly detecting miRNAs without RNA extraction, named as “Direct S-Poly(T) Plus”. This method relies on a complete denaturation of miRNA-containing protein complexes and endogenous RNase, and it is followed by a single-step, multiple-stage reaction achieving polyadenylation and reverse transcription simultaneously, during which, an elaborately designed RT primer is used. Then, the non-specific amplification of crude cDNA in quantification PCR relies on a high-activity hot-start DNA polymerase of Thermus aquaticus (Taq). With this approach, it is possible to detect miRNAs at minimal cost and time (~ 140 min). 20 μl plasma/serum could be used for approximately 100 miRNAs detection. This approach also affords higher sensitivity compared with the RNA purification-based miRNA assay, including the widely used stem-loop method and our previous method, S-Poly(T) Plus. Finally, by application of the direct S-Poly(T) Plus, seven potential biomarkers were validated from a genome-wide expression profile in plasma of colorectal cancer patients. We hope that this simple but robust protocol will enable potential circulating biomarkers to enter the clinical application very soon.

## Materials and methods

### Plasma and serum collection

Human blood samples were collected from Shenzhen People’s Hospital (Shenzhen, China) and Cancer Center of Guangzhou Medical University (Guangzhou, China). Plasma was obtained after centrifuging blood (3000*g* for 10 min at 4 °C) that has been collected in EDTA-containing tubes; Serum was drawn after centrifuging blood (3000*g* for 10 min at 4 °C) that has been kept at room temperature for 1 h and allowed to spontaneously clot. Sera were collected in the absence of anticoagulant. The plasma and sera were divided into aliquots and stored at − 80 °C. The information of participants was detailed in Additional file 1: Table S1.

### Plasma/serum preparation for miRNA measurement

To release circulating miRNAs from protein complexes, we mixed 20 μl of serum/plasma with 10 μl of 4× reaction buffer (100 mM Tris–HCl, 300 mM NaCl, 20 mM MgCl_2_, 2 mM ATP, 1 mM dNTP, pH 8.0), 10 μl RNase-free water, as well as 1 μg of proteinase K (ThermoFisher, Cat. no. 25,530,049). A 20-min incubation at 50 °C was followed by a 5-min enzyme inactivation at 95 °C. The mixture was centrifuged at 13,000*g* for 5 min at 4 °C to eliminate the protein precipitant, and then the supernatant (crude RNA) was used as the template for polyadenylation and reverse transcription.

### RT-qPCR in serum/plasma

The prepared serum/plasma supernatant was directly used as the template in one-step to complete the polyadenylation and reverse transcription (Poly(A)/RT) as in S-Poly(T) Plus protocol [25]. More specific, 10 μl of reaction mixture contains 0.5–7.5 μl of crude RNA, 1.5 μl of 4× reaction buffer, 1 μl 0.5 μM RT primer, and 1 μl Poly(A)/RT enzyme. Every microliter of Poly(A)/RT enzyme contains 1 units of Poly(A) polymerase (Enzymatics, Beverly, MA, USA) and 100 units of SuperScript III Transcriptase (Invitrogen, WY, USA). The reaction was incubated at 37 °C for 30 min, 42 °C for 30 min, and then 65 °C for 5 min. No-template control and no-reverse transcriptase control were conducted simultaneously.

Eventually, 0.5 μl of Poly(A)/RT products (crude cDNA) was amplified and detected in real-time PCR, and each qPCR (20 μl) contained 5 μl 4× qPCR Buffer, 0.5 unit hot-start Alpha Taq DNA Polymerase, 0.4 μl 10 μM forward primer, 0.4 μl 10 μM universal reverse primer, 0.5 μl 10 μM universal Taqman probe, and 0.2 μl 100× ROX reference dye. The sequence information of universal reverse primer and Taqman probe were detailed in our previous study [25]. Each reaction was performed in duplicates on ABI StepOnePlus thermal cycler at the following conditions: 95 °C for 5 min, followed by 40 cycles of 95 °C for 10 s and 60 °C for 40 s.

### RNA purification-based miRNA assay as a comparison

Extraction of total RNA, polyadenylation, reverse transcription and real-time PCR were performed using S-Poly(T) Plus method, exactly as previously detailed [25]. The other RNA purification-based miRNA assay was performed with TaqMan microRNA assay kit (Applied Biosystems), according to the manufacturer’s instructions. To make sure of directly proportional of serum/plasma inputs in RNA purification-based method and Direct S-Poly(T) Plus assay, extracted RNA was diluted as need before the next test.

### miRNA profiling

A five-step test was designed to identify potential miRNA biomarkers for colorectal cancer, including early-screening, further-screening, training, validation set-1 and validation set-2. First, 485 blood-derived miRNAs were profiled in serum samples of both colorectal cancer and healthy control and this part of the result had been published [27]. Second, comparing those in healthy cohort, miRNAs in colorectal cancer group differ by more than 1.5-fold changes on outcome were selected and confirmed with Direct S-Poly(T) Plus method. 172 plasma samples from healthy individuals and 172 from colorectal cancer patients were pooled separately and used in the further-screening. Plasma samples were collected from Shenzhen People’s Hospital (Shenzhen, China). In this step, all PCR products were detected by electrophoresis in 3.5% agarose gel, and miRNAs with nonspecific amplification were excluded. Third, miRNAs with more than 1.5-fold changes, Ct values less than 33 and without nonspecific amplification were further validated using small number of individual specimens (38 NC and 38 CRC). Ultimately, miRNAs with significant difference between colorectal cancer group and healthy group were revalidated using each individual of 106 colorectal cancer samples and 106 healthy samples. Also, potential miRNAs were confirmed with serum samples from 36 colorectal cancer patients and 36 patients from Rectum Department but without colorectal cancer. Serum samples were collected from the Cancer Center of Guangzhou Medical University (Guangzhou, China).

### Statistics

hsa-miR-93-5p was selected from 485 cancer-related miRNAs as one of the most stable miRNAs in colorectal cancer [27]. Relative quantities of miRNAs therefore were calculated using the 2^−ΔCt^ method with hsa-miR-93-5p as an endogenous normalizer. Statistical analysis was submitted to the GraphPad Prism 5. Data were shown as mean ± SE (standard error). Two-tailed Student’s test was used for statistical analysis.

## Results

### Assay design and optimization

**Fig. 1:**
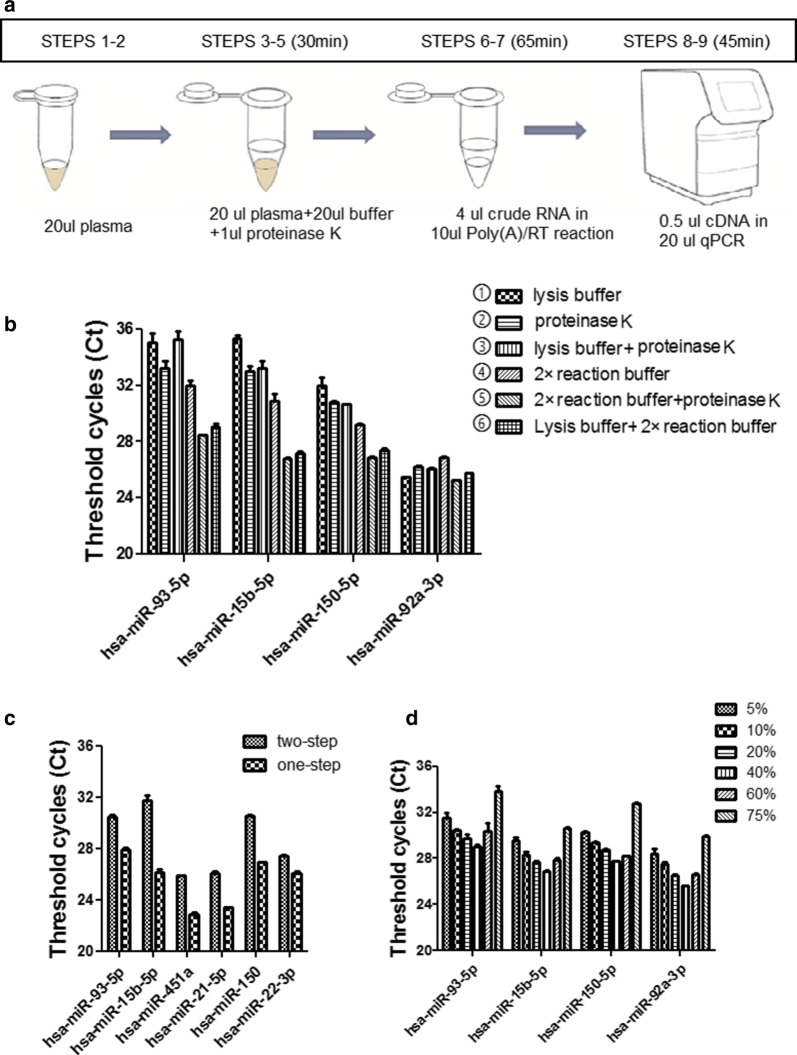
Direct S-Poly(T) Plus assay in microRNA detection. **a** Procedure of directly analyzing circulating miRNAs; **b** effects of different buffers and proteinase K in the Direct S-Poly(T) Plus assay; **c** effects of different proportions of crude RNA in Poly(A)/RT reaction; **d** sensitivity of Direct S-Poly(T) Plus when the polyadenylation and reverse transcription were performed separately (two-step) or in a single reaction (one-reaction)

This protocol describes a simple but ultrasensitive RT-qPCR assay for directly detecting microRNAs (miRNAs) without RNA purification. Using this approach, 20 μl plasma could be used for detecting approximately 100 miRNAs (with two duplicate), and it is possible to detect miRNAs with minimal cost and time (~ 140 min) (Fig. 1a). The design of the lysis step is crucial to release miRNAs from protein complexes. The general rules for this objective are the incorporation of tween 20 or proteinase K or high-temperature processing [28, 29]. We compared six combinations with different buffer or enzyme or temperature conditions as follows.

① 20 μl lysis buffer (2.5% tween-20, 50 mM Tris and 1 mM EDTA) + 20 μl plasma, 75 °C for 5 min;
② 20 μl RNase-free water + 1 μl proteinase K + 20 μl plasma, 50 °C for 20 min, and then 95 °C for 5 min;
③ 20 μl lysis buffer + 1 μl proteinase K + 20 μl plasma, 50 °C for 20 min, and then 95 °C for 5 min;
④ 20 μl 2× reaction buffer (50 mM Tris–HCl, 150 mM NaCl, 10 mM MgCl_2_, 1 mM ATP, 0.5 mM dNTP, pH 8.0) + 20 μl plasma, 75 °C for 5 min;
⑤ 20 μl 2× reaction buffer + 1 μl proteinase K + 20 μl plasma, 50 °C for 20 min, and then 95 °C for 5 min;
⑥ 10 μl 2× reaction buffer + 10 μl lysis buffer + 1 μl proteinase K + 20 μl plasma, 50 °C for 20 min, and then 95 °C for 5 min.

As a result in Fig. 1b, the effect was less satisfactory for single tween 20 (in lysis buffer) or proteinase K or combination of both (group ① ② ③). In our study, we first chose the proteinase K, because proteinase K not only released miRNA from protein complexes, but also destroyed the RNase-rich environment of plasma/serum. Furthermore, we optimized a reaction buffer for maintaining the high enzymatic activity of proteinase K, as well as being suitable for the subsequent Poly(A)/RT reaction. When 2× reaction buffer and proteinase K are combined (group ⑤), the Ct value is decreased by 0.8–6.8 compared to the combination of lysis buffer and proteinase K (group ⑥). Moreover, we proposed a hypothesis: tween 20 has a positive effect on the lysis of miRNA-coated protein complexes, as well as has a side effect on the poly(A)/RT reaction (group ⑤ and ⑥). Therefore, we recommend the condition of group ⑤ as the optimal condition in our direct RT-qPCR assay.

To optimize our direct quantification of circulating miRNAs assay, we tested effects of different proportions of supernatant inputs (crude RNA) in polyadenylation reaction and reverse transcription. The plasma from a same sample was used as the initial material. When the volume percentage of crude RNA ranged from 5 to 40%, Ct values were continuously decreased by 2.4–2.8 Ct values. However, when 60% of crude RNA were used in the corresponding reaction, the decrease of a trend of Ct values was reversed; therefore, the optimization of material inputs was defined as 40% of crude RNA (Fig. 1c).

Like that in our previous RNA purification-based miRNA assay, the S-Poly(T) Plus method, when polyadenylation and reverse transcription in the S-Poly(T) method are carried out simultaneously, Ct values of miRNA decreased by 1.3–3.6 units in Direct S-Poly(T) Plus assay. Therefore, this procedure also minimized the reaction time and serum/plasma inputs, and improved the sensitivity in Direct S-Poly(T) Plus assay (Fig. 1d).

### Plasma vs. serum

**Fig. 2:**
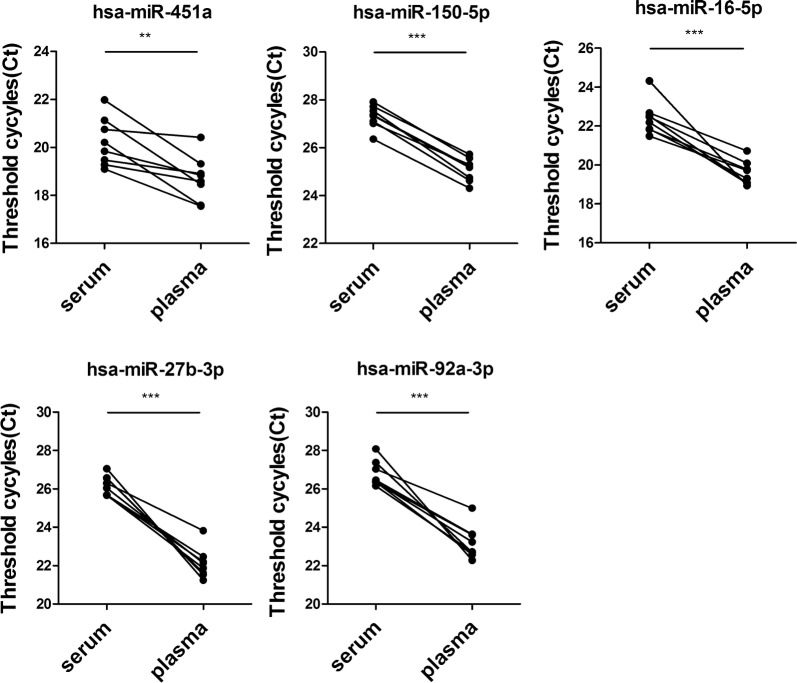
Measurements of five miRNAs (hsa-miR-451a, hsa-miR-150-5p, has-miR-16-5p, hsa-miR-27b-3p and hsa-miR-92a-3p) with Direct S-Poly(T) Plus method using serum and plasma as initial materials. Each volume of plasma and corresponding serum came from a same healthy donor. **p < 0.01, ***p < 0.001

Plasma and serum both are cell-free supernatant. To be more specific, plasma is obtained after centrifuging blood that has been collected with anticoagulant, while serum is obtained after centrifuging blood without an anticoagulant that was left to spontaneously clot. In this study, plasma and serum had been proved to be usable in the direct quantification of circulating miRNAs. However, when an equal volume of initial serum and corresponding plasma were obtained from a same healthy donor, the Ct values of miRNAs as assayed with plasma were significantly smaller than those with serum (Fig. 2). This result was reconfirmed using extracted RNA and higher relative expression levels of the plasma were detected in S-Poly(T) Plus assay than those of the serum (Additional file 2: Figure S1). Therefore, miRNAs were more abundant in plasma than those in serum. Unless stated, in the following trials, plasma was used as initial materials.

### Nonspecific amplification

**Fig. 3:**
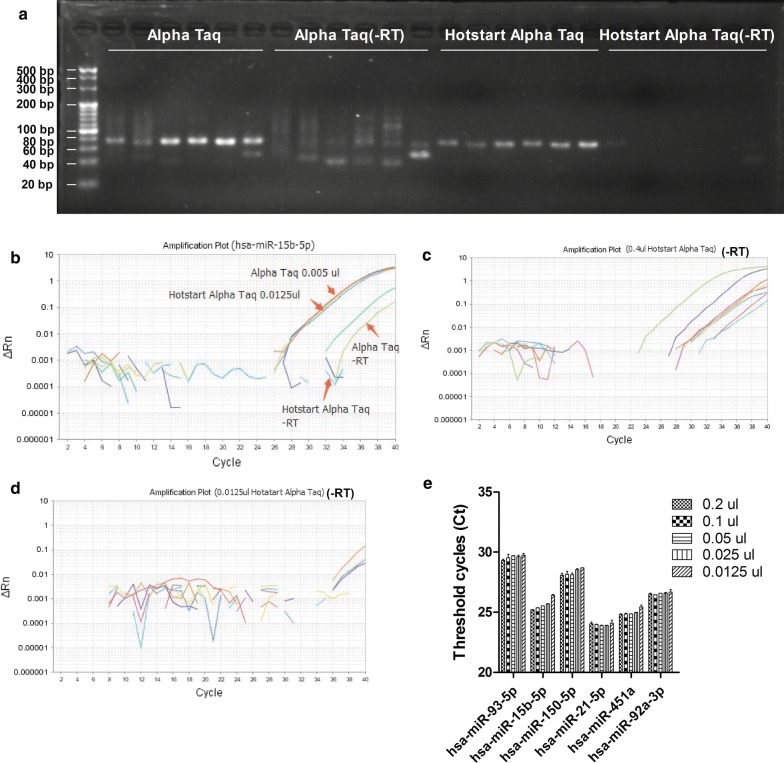
Optimization of the Direct S-Poly(T) Plus assay. **a** Reducing effects of hotstart Alpha Taq polymerase on the non-specific amplification, RT-qPCR products of five miRNAs (hsa-miR-93-5p, hsa-miR-15b-5p, hsa-miR-150-5p, hsa-miR-21-5p, hsa-miR-451a and hsa-miR-92a-3p) were conducted electrophoresis, -RT: no-reverse transcriptase control; **b** amplification plot of hsa-miR-15b-5p with normal and hot-start Alpha Taq polymerase; **c** amplification plot of hsa-miR-93-5p, hsa-miR-15b-5p, hsa-miR-150-5p, hsa-miR-21-5p, hsa-miR-451a and hsa-miR-92a-3p without reverse transcriptase using Alpha Taq polymerase; **d** amplification plot of hsa-miR-93-5p, hsa-miR-15b-5p, hsa-miR-150-5p, hsa-miR-21-5p, hsa-miR-451a and hsa-miR-92a-3p without reverse transcriptase using hot-start Alpha Taq polymerase; **e** correlation of different amount of hot-start Alpha Taq polymerase inputs to the threshold cycle (Ct)

Nonspecific amplification was one of the major problems in miRNA detection. Without RNA purification, it is possible to incorporate a mass of genome DNA as a template; more unwanted DNA synthesis can occur during the qPCR set-up. We compared several kinds of DNA polymerase (Additional file 3: Figure S2) and found that Alpha Taq polymerase (VitaNavi, St. Louis USA) maintaining the high enzymatic activity in the direct PCR assay. We test hsa-miR-93-5p, hsa-miR-15b-5p, hsa-miR-150-5p, hsa-miR-21-5p, hsa-miR-451a and hsa-miR-92a-3p with Alpha Taq polymerase; however, there was still some amplification in the negative control (-RT, without reverse transcriptase) (Fig. 3a). One of the useful ways to reduce nonspecific DNA synthesis is a “hot start”, wherein DNA synthesis is reduced or prevented at ambient temperature prior to thermal cycling [30], and one of the most effective ways of arranging a hot start is using anti-taq antibodies, which could reduce the DNA polymerase activity, but being thermolabile, release the enzyme at PCR cycling temperatures [31]. The data showed that it is critical for reducing nonspecific amplification with a hot-start in the qPCR assay (Fig. 3a–d). It was proved that hot-start Alpha Taq was highly-active, and 0.0125 μl was enough for 20 μl qPCR reaction (Fig. 3e).

### Sensitivity and dynamic range

**Fig. 4:**
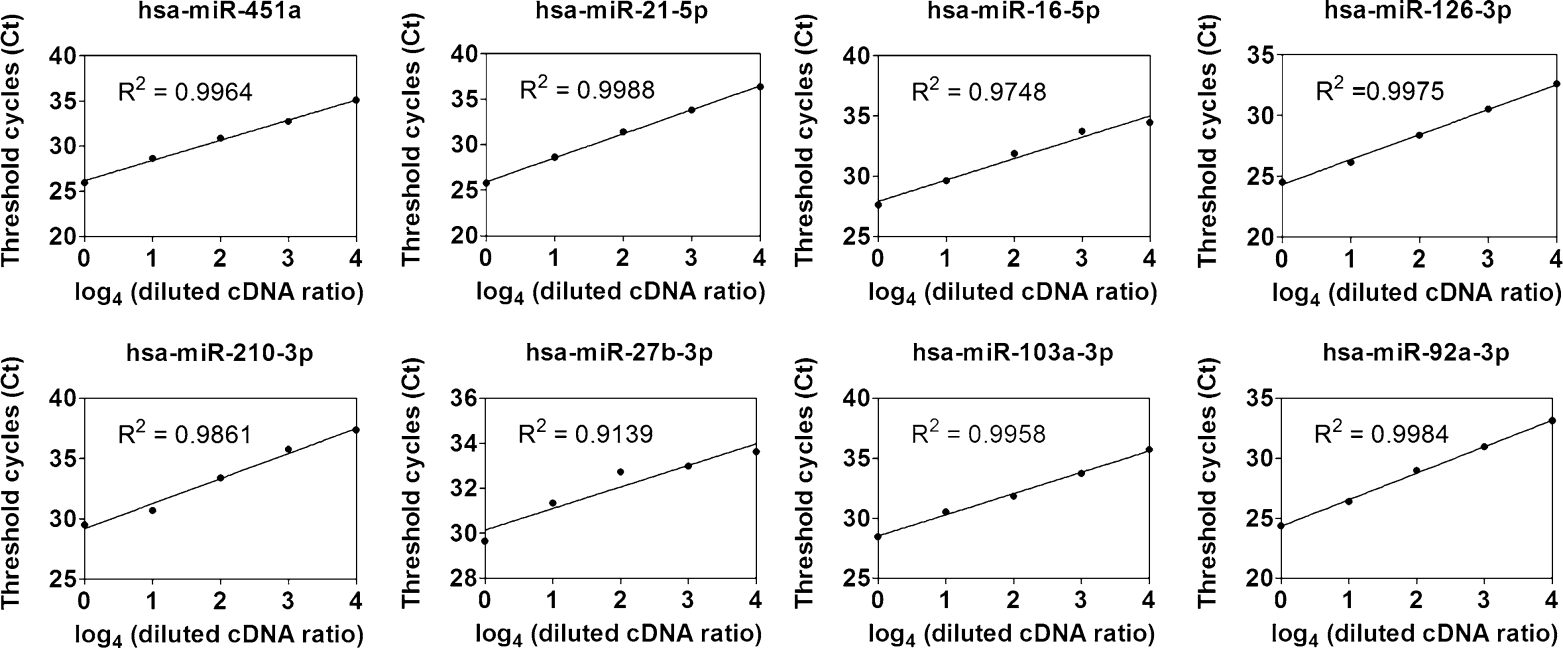
Dynamic range and sensitivity of the Direct S-Poly(T) Plus method. Expression levels of **a** hsa-miR-451a, **b** hsa-miR-21-5p, **c** hsa-miR-16-5p, **d** hsa-miR-126-3p, **e** hsa-miR-210-3p, **f** hsa-miR-27b-3p, **g** hsa-miR-103a-3p and **h** hsa-miR-92a-3p from plasma samples with 1–256 times cDNA dilutions

To demonstrate the sensitivity of the direct quantification method for measuring miRNA, the crude cDNAs were diluted 4 times every time and expression levels of eight miRNAs (hsa-miR-451a, hsa-miR-21-5p, hsa-miR-126-3p, hsa-miR-92a-3p, hsa-miR-210-3p, hsa-miR-27b-3p, hsa-miR-103a-3p and hsa-miR-92a-3p) were determined. The plasma mixture sample was used in this test, which made of 30 samples from healthy donors. The results showed that the correlation coefficients (R^2^) produced by quantification of miRNAs direct in plasma ranged from 0.9139 (hsa-miR-27b-3p) to 0.9988 (hsa-miR-21-5p) with 1–256 times cDNA dilutions (Fig. 4). In Direct S-Poly(T) Plus method, 20 μl plasma could be used for 100 μl cDNA preparation, and then for 200 miRNAs detection (without duplication). In that sense, 0.1–0.0003 μl (cDNA was diluted from 0 to 256 times) of initial plasma inputs were sufficient for single miRNA measurement on average.

### Comparison of the direct RT-qPCR assay with stem-loop and S-Poly(T) Plus method

**Fig. 5:**
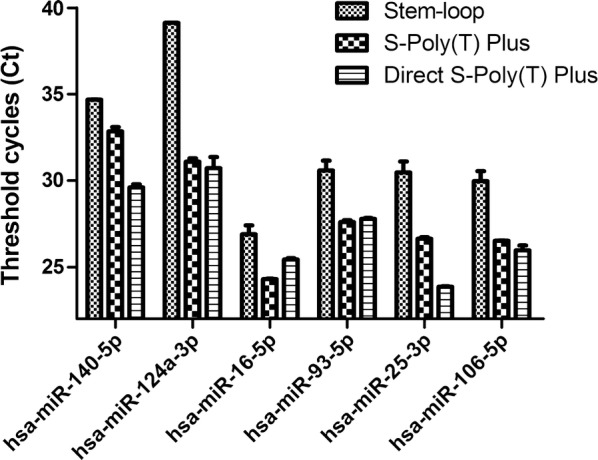
Comparison of the Direct S-Poly(T) Plus assay in the current study with stem-loop and S-Poly(T) Plus method. Threshold cycle (Ct) values were produced by three quantification assays; extracted RNA was used in stem-loop and S-poly(T) plus assay; plasma was used in the Direct S-Poly(T) Plus assay as initial materials, respectively

To determine the efficiency of the direct quantification of miRNA assay, we compared it with the other two miRNA assays, widely-used stem-loop method and our previous assays, S-Poly(T) Plus method and the extracted RNA was used as the template in the two methods. To make sure of an equal volume of initial plasma in reverse transcription, the extracted RNA was diluted. Usually, the purified RNA was extracted from 100 μl of plasma/serum and dissolved into 25 μl; and approximately 40 μl crude RNA was obtained from 20 μl plasma. Therefore, the purified RNA used was diluted 8 times before reverse transcription. Extraction of total RNA, polyadenylation, reverse transcription and real-time PCR were performed using S-Poly(T) Plus method, exactly as previously detailed [25]. The stem-loop method was performed with TaqMan microRNA assay kit (Applied Biosystems), according to the manufacturer’s instructions. hsa-miR-140-5p, hsa-miR-124a-3p, hsa-miR-16-5p, hsa-miR-93-5p, hsa-miR-25-3p and hsa-miR-106-5p were examined in the three methods, respectively. As shown in Fig. 5, except that Ct values of hsa-miR-16-3p (25.43) and hsa-miR-93-5p (27.78) were slightly higher than those in the S-poly(T) Plus (24.29 and 27.60, respectively), all Ct values produced by direct RT-qPCR were the smallest in the three methods. The sensitivity of Direct S-Poly(T) Plus is 2.7–343-fold higher (1.5–8.4 Ct value difference) than that of stem-loop method.

### miRNA expression profiling in colorectal cancer

**Fig. 6:**
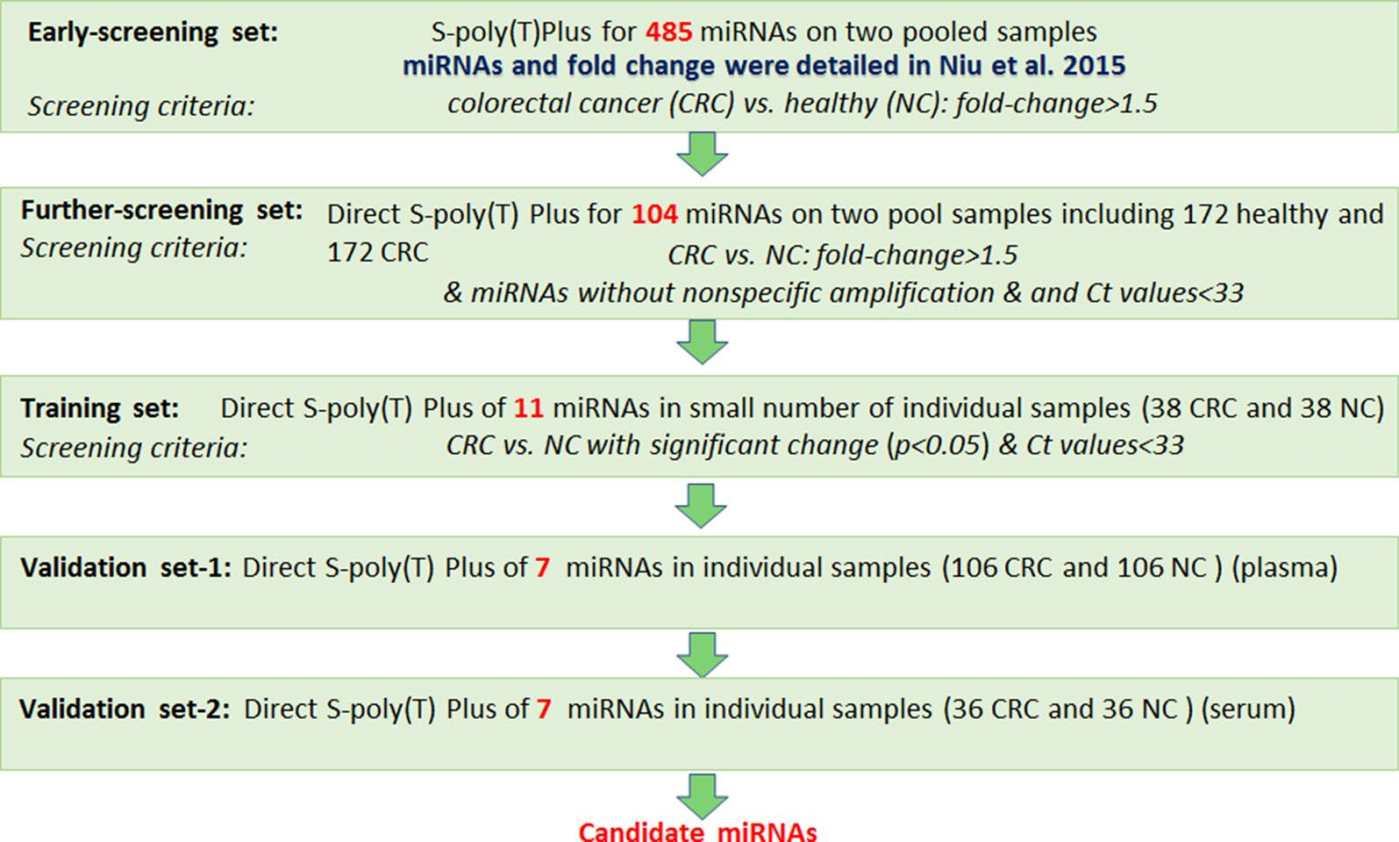
Overview of the screening process. The flow diagram illustrates the five steps of miRNAs selection as potential biomarkers from 485 miRNAs in colorectal cancer (CRC)

**Fig. 7:**
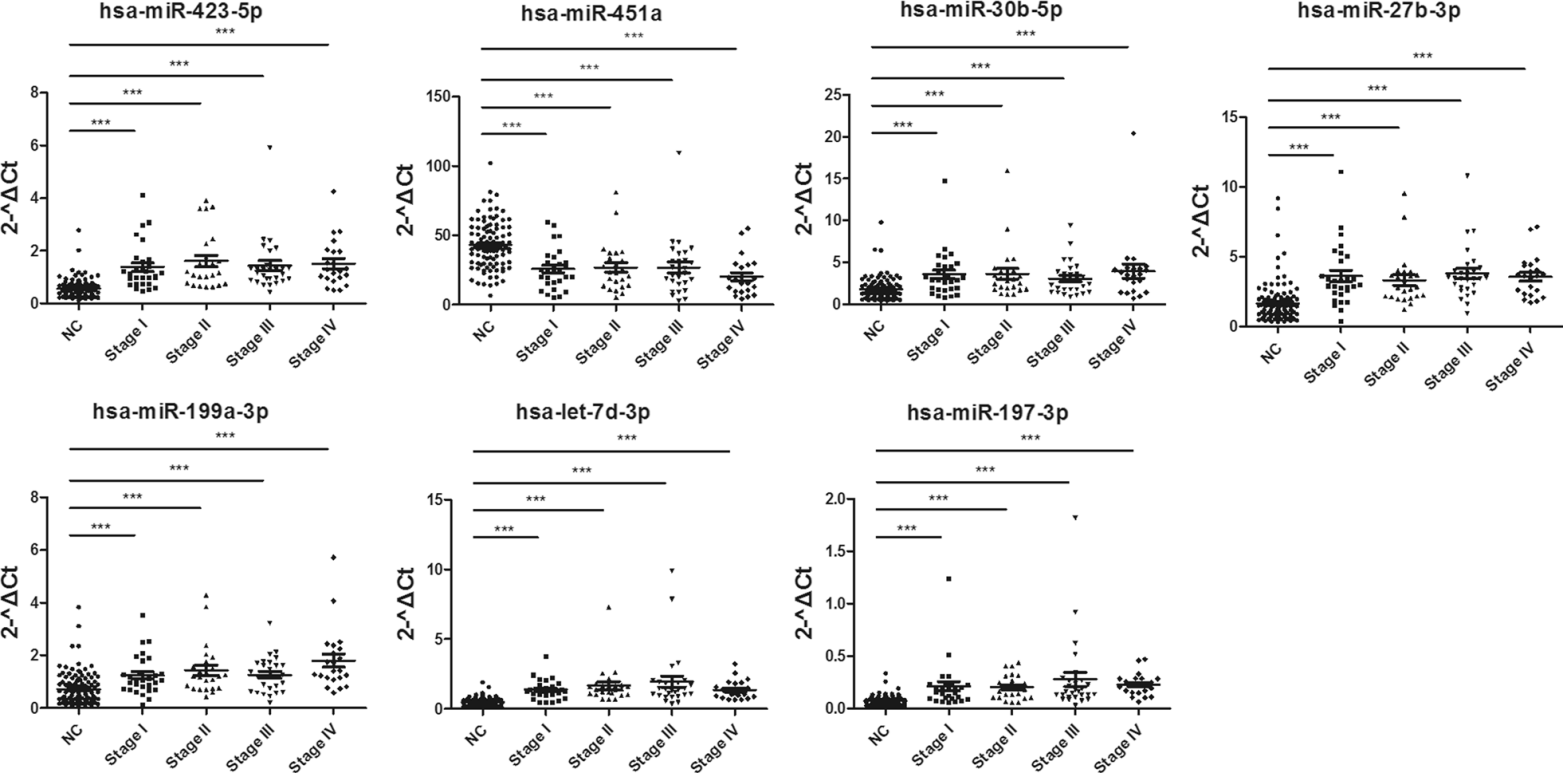
Differentially expressed miRNAs in the plasma of colorectal cancer patients and healthy individuals with the Direct S-Poly(T) Plus assay. Data are shown as mean ± SE, **p < 0.01, ***p < 0.001

In our previous study, 485 human blood-derived miRNA had been validated between colorectal cancer patients and healthy individuals, and three miRNAs (hsa-miR-93-5p, hsa-miR-25-3p and hsa-miR-106b-5p) were identified as suitable endogenous references [27]. Based on these profiling results, we conducted another five-step test to select biomarkers of colorectal cancer, and the whole study flow chart was depicted as in Fig. 6. 104 miRNAs with a significant difference were selected from 485 miRNA-profile and were detected with Direct S-Poly(T) Plus (further-screening) (Additional file 4: Figure S3). Electrophoresis of RT-qPCR product was conducted to detect nonspecific amplification; we selected 11 miRNAs with at least 1.5-fold change, Ct values less than 33 and without nonspecific amplification. These 11 miRNAs were validated in a small number of individual specimens (training set) (Additional file 5: Figure S4); miRNAs with significant difference (*p *< *0.05*) were revalidated with 106 colorectal cancer cases and 106 healthy controls, and expression levels of all seven miRNAs significantly differed between colorectal cancer patients and healthy controls (validation set-1). Besides, seven miRNAs could discriminate colorectal cancer stage I from healthy individuals, showing exciting prospects for early diagnosis and prognosis (Fig. 7). To test the accuracy of potential biomarkers and Direct S-Poly(T) Plus method, RT-qPCR products of seven miRNAs were cloned to the vector and sequenced, and sequencing results were depicted in Additional file 6: Figure S5. Therefore, seven miRNAs were identified as potential biomarkers in colorectal cancer, and they were hsa-miR-423-5p, hsa-miR-451a, hsa-miR-30b-5p, hsa-miR-27b-3p, hsa-miR-199a-3p, hsa-let-7d-3p and hsa-miR-197-3p.

In addition, seven potential miRNA biomarkers were confirmed with serum samples from 36 colorectal cancer patients and 36 patients from Rectum Department but without colorectal cancer, and these serum samples were collected from Cancer Center of Guangzhou Medical University (Guangzhou, China). The result showed change trends of seven miRNAs were same as in validation set-1, proving that our selection and validation procedure are reliable. However, hsa-miR-451a and hsa-miR-30b-5p changed not significantly between colorectal cancer and control group in serum samples (validation set-2) (Additional file 7: Figure S6), and the possible reasons could be that miRNAs were less abundant in serum than those in plasma (Fig. 2 and Additional file 2: Figure S1); control individuals might be not healthy but with some other anorectal diseases.

**Fig. 8:**
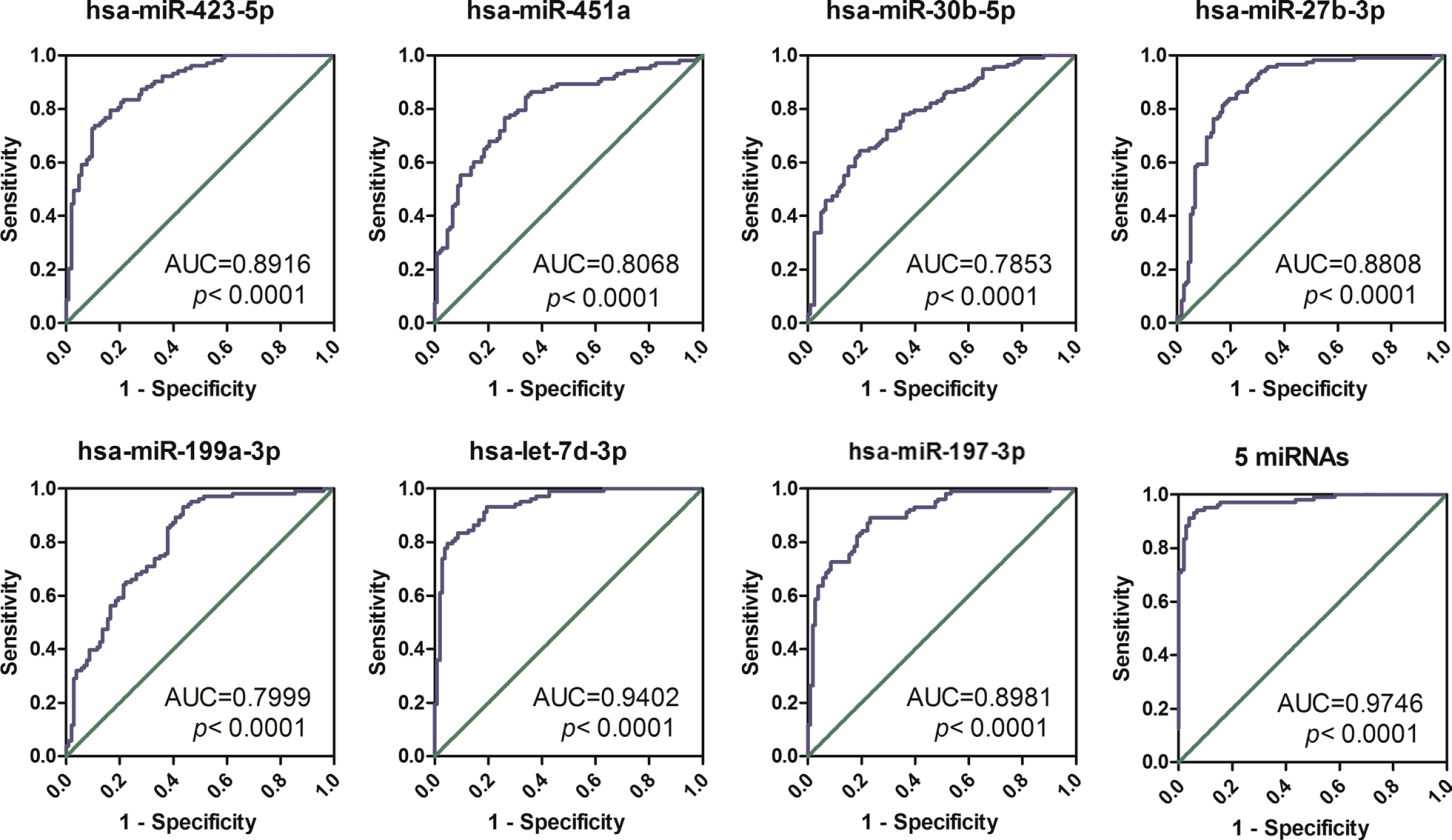
Receiver operating characteristic curves (ROC) of seven individual miRNAs as potential biomarkers in colorectal cancer, as well as five miRNAs as one combined biomarker

In ROC curve analysis, single miRNA promised satisfactory discrimination with area under ROC curve (AUC) ranging from 0.79 to 0.94 (*p* value < 0.001). When conducted multivariate logistic regression analysis, five miRNAs (hsa-miR-451a, hsa-miR-30b-5p, hsa-miR-27b-3p, hsa-miR-199a-3p and hsa-let-7d-3p) entered as variables (*p *< 0.05). We combined these five miRNAs as a single biomarker and yielded a very high AUC of 0.97 (Fig. 8) with a sensitivity of 95.1% and a specificity of 90.3%.

## Discussion

The Direct S-Poly(T) Plus quantification protocol described here is simple, efficient and sensitive for measuring circulating miRNAs without RNA extraction. Usually, trizol reagent is formulated for total RNA isolation, however, it is unavoidable to have some of the miRNAs lost due to incomplete protein denaturation or incomplete RNA precipitation and recovery. More importantly, it is very tedious and time-consuming for processing large numbers of samples, which limits its clinical application. Strategies for direct RT-qPCR analysis in the measurement of circulating miRNAs had been proposed in cell lysates [32], serum [28] or plasma [29]. Comparing to these approaches, we have made more efforts to improve the sensitivity of the method in this study. With Direct S-Poly(T) Plus assay, 20 μl plasma could be used for detecting approximately 100 miRNAs (with two duplicate), and it is possible to detect single miRNA with 0.0003 μl initial plasma inputs (Fig. 4), which is much less than that in the reports of Asaga et al. [28] (0.625 μl) and Zhao et al. [29] (0.02 μl). The sensitivity of direct S-Poly(T) Plus relies on: first, a complete denaturation of miRNA-containing protein complexes and endogenous RNase. According to the literature, this success may in part reflect the incorporation of tween 20 or proteinase K [28, 29]. In our study, we discovered that miRNAs were more easily detected in the proteinase K digestion of plasma (Fig. 1b). This result would be explained by that proteinase may be more effective for the digestion of protein complexes, and destroy the RNase-rich environment of plasma/serum for decreasing degradation of miRNA; second, an elaborately designed RT primer, which consists of an oligo(dT)_11_ sequence and six miRNA-specific bases, thus provides higher binding strength and thermodynamic stability between miRNA template and RT primer [12, 25]; Third, a single-step, multiple-stage reaction achieving polyadenylation and reverse transcription simultaneously [25]; fourth, a high-activity hot-start DNA polymerase Taq for the crude cDNA. Comparing to RNA purification-based assays, the sensitivity of Direct S-Poly(T) Plus assay was 2.7–343-fold higher than that of the widely used stem-loop method, and comparable with the previous version, S-Poly(T) plus method.

The direct identification of circulating miRNAs may impact the development of specific miRNAs as biomarkers. We also made lots of efforts on improving the applicability of Direct S-Poly(T) Plus and the performance of miRNA biomarkers in clinic cases. In previous literature, only several miRNAs were detected to determine the usability and sensitivity of direct RT-qPCR assay [28, 29]. In our study, hundreds of miRNAs had been tested using Direct S-Poly(T) Plus method and part of results were verified with RNA purification-based method, and then potential biomarkers with high AUC and sensitivity were validated. Besides, data normalization is a challenge for analysis for circulating miRNA, especially in direct RT-qPCR assay. Spiked-in RNAs, such as cel-miR-39, cel-miR-54, and cel-miR-238, could only monitor the efficiency of RNA purification or RT as a class of exogenous references. However, these non-protein-complexes-coated exogenous references were specifically destabilized in Rnase-rich plasma/serum. Suitable endogenous reference genes could be expressed constitutively and the expression levels should not be affected by biological change, disease or treatment. In our previous study, we identified three endogenous references (hsa-miR-93-5p, hsa-miR-25-3p and hsa-miR-106b-5p) out of from 485 blood-derived miRNAs, which could stably express in different cohorts of plasma samples of colorectal cancer and healthy donor [27]. More interestingly, three miRNAs validated from 485 miRNAs are derived from a single primary transcript, indicting the cluster may be highly conserved in colorectal cancer. In this study, we used one of them, hsa-miR-93-5p as reference, and then seven miRNAs (hsa-miR-423-5p, hsa-miR-451a, hsa-miR-30b-5p, hsa-miR-27b-3p, hsa-miR-199a-3p, hsa-let-7d-3p and hsa-miR-423-5p) were validated as potential biomarkers in colorectal cancer. Importantly, these seven miRNAs could discriminate colorectal cancer stage I from healthy individuals, which generates exciting prospects for early diagnosis and prognosis.

Advances in miRNA biomarkers have generated a large number of disease markers with potential clinical values [33–37]. It was even reported that miRNA expression analyses in plasma samples collected 1–2 years before the onset of lung cancer, at the time of CT detection, resulting in the generation of miRNA signatures with strong predictive potential [36]. We also have validated 11 miRNA biomarkers of non-small cell lung cancer from genome-wide expression profile, and these miRNAs are reliable in different hospital samples, pooled or individual samples [38]. However, none of these published results have been applied in clinic until today. The main reason could be lacking a very simple but robust standardized execution of miRNA measurements with clinical application value. Direct S-Poly(T) Plus assay could minimize human and mechanical errors and reduce time and cost. Using this approach, a detection report could be obtained in 2–3 h and only 20 μl plasma is enough for a panel of miRNAs.

## Conclusions

Therefore, it is convenient and economical as a predictive or additional diagnose tool in the hospital. We will further test previously validated biomarkers of colorectal cancer, non-small cell lung cancer and other diseases with more samples from different sources. In the future, we aim to establish more collaboration with hospitals and test rapid diagnostic trial kits in colorectal cancer and non-small cell lung cancer. We hope that this simple and robust protocol may have a strong impact on the development of specific miRNAs as biomarkers in the clinic.

## Supplementary information

**Additional file 1: Table S1.** Characteristics of patients and healthy controls enrolled in this study.

**Additional file 2: Figure S1.** Expression levels of has-miR-451a, has-miR-150-5p, has-miR-27b-3p and has-miR-92a-3p using purified RNA as template with the S-Poly(T) Plus method . Each volume of plasma and corresponding serum were from a same healthy donor. miRNA levels were normalized to spiked-in cel-miR-54-5p. Data are shown as means ± SE, ***p < 0.001.

**Additional file 3: Figure S2.** Comparison of four kinds of DNA polymerase in the Direct S-Poly(T) Plus assay. Plasma was used as template. Alpha taq, Superm taq and Omni taq were purchased from VitaNavi company (VitaNavi, St. Louis USA) and HSSM taq were purchased from Geneup company (Geneup, Shenzhen, China).

**Additional file 4: Figure S3.** Expression pattern of 104 miRNAs. Heatmap depicted miRNAs differentially expressed between healthy (NC) and colorectal cancer (CRC) pooled samples. miRNAs were detected with Direct S-Poly(T) Plus method.

**Additional file 5: Figure S4.** Differentially expressed miRNAs in 38 plasma samples of colorectal cancer patients and 38 healthy individuals with the Direct S-Poly(T) Plus. Data are shown as means ± SE, *p < 0.05, ***p < 0.001, ns, not significant.

**Additional file 6: Figure S5.** The sequencing information of 7 potential miRNA biomarkers and reference miRNA. A. Alignment of mature miRNAs and sequencing results; B. Sanger sequencing peak and quality; C. Schematic diagram of cloning RT-qPCR products to plasmid vectors.

**Additional file 7: Figure S6.** Differentially expressed miRNAs in 36 serum samples of colorectal cancer patients and 36 controls with the Direct S-Poly(T) Plus. Serum samples were collected from Cancer Center of Guangzhou Medical University (Guangzhou). Data are shown as means ± SE, **p < 0.01, ***p < 0.001, ns, not significant.

## Data Availability

We will make available all data and materials.

## Abbreviations

miRNA: microRNA
CRC: colorectal cancer
RT-qPCR: reverse-transcription quantitative real-time PCR
Taq: thermus aquaticus
Poly(A)/RT: polyadenylation and reverse transcription
