# Supplementary material for: Direct S-Poly(T) Plus assay in quantification of microRNAs without RNA extraction and its implications in colorectal cancer biomarker studies
Source: J Transl Med. 2019 Sep 23;17:316. doi: 10.1186/s12967-019-2061-6 (PMC6757382; doi:10.1186/s12967-019-2061-6)
Supplement: Supplementary file 1 — Additional file 1: Table S1. Characteristics of patients and healthy controls enrolled in this study. [file 12967_2019_2061_MOESM1_ESM.pdf]

Additional file 1: Table S1. Characteristics of patients and healthy controls enrolled in this study.

A. Plasma samples in study of miRNA biomarker selection collected from Shenzhen People's Hospital (Shenzhen, China).

colorectal cancer

number	age	gender	TNM
1	57	male	I
2	33	female	I
3	55	male	I
4	54	male	I
5	46	male	I
6	56	female	I
7	58	female	I
8	62	female	I
9	79	female	I
10	57	male	I
11	77	male	I
12	61	female	I
13	49	female	I
14	77	female	I
15	61	female	I
16	49	female	I
17	63	female	I
18	56	female	I
19	52	female	I
20	65	male	I
21	69	female	I
22	63	male	I
23	68	female	I
24	50	female	I
25	52	female	I
26	72	male	I
27	77	male	I
28	60	male	I
29	71	female	I
30	60	female	I
31	43	female	I
32	76	male	I
33	65	male	I
34	79	female	I
35	75	female	I
36	60	male	I
37	57	female	I
38	60	male	I
39	61	male	I
40	70	male	I
41	67	female	I
42	57	female	I
43	51	female	I
44	73	female	I A

healthy

number	gender	age
1	male	57
2	female	36
3	male	55
4	male	54
5	male	48
6	female	58
7	female	59
8	female	62
9	female	77
10	male	44
11	female	81
12	female	55
13	male	49
14	female	61
15	female	81
16	female	62
17	female	58
18	female	62
19	male	64
20	male	39
21	male	57
22	male	65
23	male	54
24	male	56
25	male	81
26	male	57
27	female	58
28	female	57
29	female	60
30	male	73
31	male	67
32	female	66
33	female	73
34	female	68
35	male	58
36	male	67
37	female	59
38	male	55
39	female	66
40	male	61
41	female	55
42	female	59
43	female	58
44	female	59

45	38	female	Ⅰ A
46	57	male	Ⅰ A
47	61	female	Ⅰ A
48	70	male	Ⅰ A
49	76	female	Ⅰ A
50	56	female	Ⅰ A
51	40	male	Ⅰ B
52	74	female	Ⅰ B
53	65	male	Ⅰ B
54	41	female	Ⅱ
55	38	female	Ⅱ
56	81	female	Ⅱ
57	63	female	Ⅱ
58	58	female	Ⅱ
59	65	male	Ⅱ
60	58	female	Ⅱ
61	58	female	Ⅱ
62	46	male	Ⅱ A
63	42	male	Ⅱ A
64	63	male	Ⅱ A
65	61	female	Ⅱ A
66	56	male	Ⅱ A
67	57	male	Ⅱ A
68	88	male	Ⅱ A
69	59	male	Ⅱ A
70	77	female	Ⅱ A
71	70	male	Ⅱ A
72	36	female	Ⅱ B
73	32	male	Ⅱ B
74	38	female	Ⅱ B
75	36	female	Ⅱ B
76	55	male	Ⅱ B
77	53	male	Ⅱ B
78	40	female	Ⅱ B
79	66	male	Ⅱ B
80	63	male	Ⅱ B
81	60	male	Ⅱ B
82	64	female	Ⅱ B
83	56	male	Ⅱ B
84	68	male	Ⅱ B
85	66	female	Ⅱ B
86	76	male	Ⅱ B
87	66	male	Ⅱ B
88	65	male	Ⅱ B
89	58	female	Ⅱ B
90	80	female	Ⅱ C
91	65	female	Ⅱ C
92	82	female	Ⅱ C
93	79	female	Ⅱ C

45	female	44
46	male	63
47	male	58
48	male	60
49	male	55
50	male	54
51	male	46
52	female	72
53	female	78
54	female	55
55	female	46
56	female	56
57	female	57
58	male	56
59	female	56
60	female	56
61	male	56
62	male	49
63	male	46
64	male	64
65	female	62
66	female	56
67	male	54
68	female	55
69	female	58
70	male	61
71	male	64
72	female	37
73	male	37
74	female	46
75	female	37
76	male	55
77	male	53
78	female	48
79	male	59
80	male	55
81	female	69
82	male	55
83	male	56
84	male	54
85	male	78
86	male	71
87	female	75
88	male	59
89	male	67
90	female	61
91	female	70
92	female	70
93	female	83

94	58	female	ⅢA
95	37	male	ⅢA
96	33	male	ⅢA
97	53	male	ⅢA
98	78	female	ⅢA
99	54	male	ⅢA
100	69	male	ⅢA
101	81	male	ⅢA
102	73	male	ⅢA
103	66	male	ⅢA
104	50	female	ⅢA
105	66	male	ⅢA
106	62	male	ⅢA
107	70	male	ⅢA
108	62	male	ⅢA
109	37	female	ⅢB
110	74	female	ⅢB
111	32	male	ⅢB
112	63	male	ⅢB
113	65	male	ⅢB
114	85	female	ⅢB
115	66	female	ⅢB
116	66	male	ⅢB
117	56	female	ⅢB
118	63	male	ⅢB
119	45	female	ⅢB
120	57	male	ⅢB
121	62	male	ⅢB
122	49	female	ⅢB
123	33	male	ⅢC
124	37	female	ⅢC
125	27	male	ⅢC
126	43	male	ⅢC
127	54	male	ⅢC
128	45	male	ⅢC
129	35	male	ⅢC
130	65	female	ⅢC
131	72	male	ⅢC
132	65	female	ⅢC
133	60	female	ⅢC
134	50	female	ⅢC
135	57	male	ⅢC
136	57	female	ⅢC
137	36	female	Ⅳ
138	45	male	Ⅳ
139	60	female	Ⅳ
140	47	male	Ⅳ
141	49	female	Ⅳ

94	female	60
95	male	46
96	male	42
97	male	53
98	female	55
99	female	47
100	female	60
101	female	66
102	female	82
103	male	79
104	male	61
105	female	73
106	male	57
107	female	57
108	male	53
109	female	43
110	female	72
111	male	44
112	female	66
113	female	63
114	female	65
115	female	60
116	male	58
117	male	60
118	male	57
119	female	58
120	female	55
121	male	62
122	male	52
123	male	43
124	female	43
125	male	34
126	male	47
127	male	54
128	male	47
129	male	83
130	male	60
131	male	54
132	female	60
133	female	60
134	male	62
135	male	55
136	male	54
137	female	42
138	male	48
139	female	61
140	female	75
141	female	76

142	35	male	Ⅳ
143	38	female	Ⅳ
144	68	male	Ⅳ
145	77	male	Ⅳ
146	60	female	Ⅳ
147	57	female	ⅣA
148	30	male	ⅣA
149	66	male	ⅣA
150	63	male	ⅣA
151	58	female	ⅣA
152	54	male	ⅣA
153	57	female	ⅣA
154	53	female	ⅣA
155	65	female	ⅣA
156	65	male	ⅣA
157	64	female	ⅣA
158	68	male	ⅣA
159	72	female	ⅣA
160	60	male	ⅣA
161	59	male	ⅣA
162	45	female	ⅣA
163	32	female	ⅣB
164	63	male	ⅣB
165	52	male	ⅣB
166	72	female	ⅣB
167	55	male	ⅣB
168	55	male	ⅣB
169	64	male	ⅣB
170	60	female	ⅣB
171	63	male	ⅣB
172	57	male	ⅣB

142	male	54
143	male	59
144	female	67
145	male	65
146	male	54
147	female	59
148	male	35
149	male	57
150	male	58
151	female	64
152	female	56
153	male	62
154	female	61
155	female	66
156	female	79
157	male	56
158	male	65
159	male	59
160	female	58
161	male	62
162	female	55
163	female	27
164	male	65
165	male	52
166	female	69
167	male	59
168	female	63
169	male	83
170	female	56
171	female	59
172	male	57

B. Serum samples in study of miRNA biomarker confirmation collected from Cancer Center of Guangzhou Medical University (Guangzhou, China).

patients with colorectal cancer

number	age	gender	TNM
1	42	female	Ⅰ
2	63	female	Ⅰ
3	55	male	Ⅰ
4	43	male	Ⅰ
5	51	male	Ⅰ
6	72	female	Ⅰ
7	30	female	Ⅱ
8	26	female	Ⅱ
9	58	female	ⅡA
10	67	female	ⅡA
11	35	female	ⅡA

patients from Rectum Department but without colorectal cancer

number	age	gender
1	46	female
2	49	female
3	47	male
4	37	male
5	53	female
6	39	male
7	34	female
8	39	female
9	51	female
10	39	female
11	54	female

12	57	male	ⅡA
13	52	male	ⅡA
14	43	female	ⅡB
15	61	male	ⅡB
16	71	female	ⅡB
17	67	male	ⅡB
18	39	female	ⅡB
19	44	male	ⅡB
20	47	male	ⅡB
21	38	female	ⅡC
22	50	male	ⅢB
23	60	female	ⅢB
24	56	male	ⅢB
25	51	female	ⅢB
26	42	male	ⅢC
27	45	male	ⅢC
28	53	male	ⅢC
29	69	female	ⅢC
30	60	male	ⅢC
31	61	male	ⅢC
32	62	male	ⅢC
33	57	female	Ⅳ
34	57	male	Ⅳ
35	63	male	Ⅳ
36	56	female	ⅣB

12	54	female
13	41	female
14	44	female
15	53	female
16	37	male
17	38	male
18	35	female
19	33	female
20	36	male
21	36	male
22	34	male
23	44	female
24	44	female
25	44	female
26	45	female
27	46	male
28	44	male
29	45	male
30	41	male
31	48	female
32	46	female
33	49	female
34	34	female
35	57	male
36	50	male

C.Samples in test of plasma vs. serum collected from Shenzhen People's Hospital (Shenzhen, China).

healthy

number	gender	age
1	male	46
2	male	42
3	female	40
4	female	43
5	female	28
6	male	46
7	female	53
8	female	46
9	male	57
10	female	52
