# Supplementary material for: Direct S-Poly(T) Plus assay in quantification of microRNAs without RNA extraction and its implications in colorectal cancer biomarker studies
Source: J Transl Med. 2019 Sep 23;17:316. doi: 10.1186/s12967-019-2061-6 (PMC6757382; doi:10.1186/s12967-019-2061-6)

Additional file 2: Figure S1. Expression levels of has-miR-451a, has-miR-150-5p, has-miR-27b-3p and has-miR-92a-3p using purified RNA as template with the S-Poly(T) Plus method . Each volume of plasma and corresponding serum were from a same healthy donor. miRNA levels were normalized to spiked-in cel-miR-54-5p. Data are shown as means ±SE, ****P*<0.001.

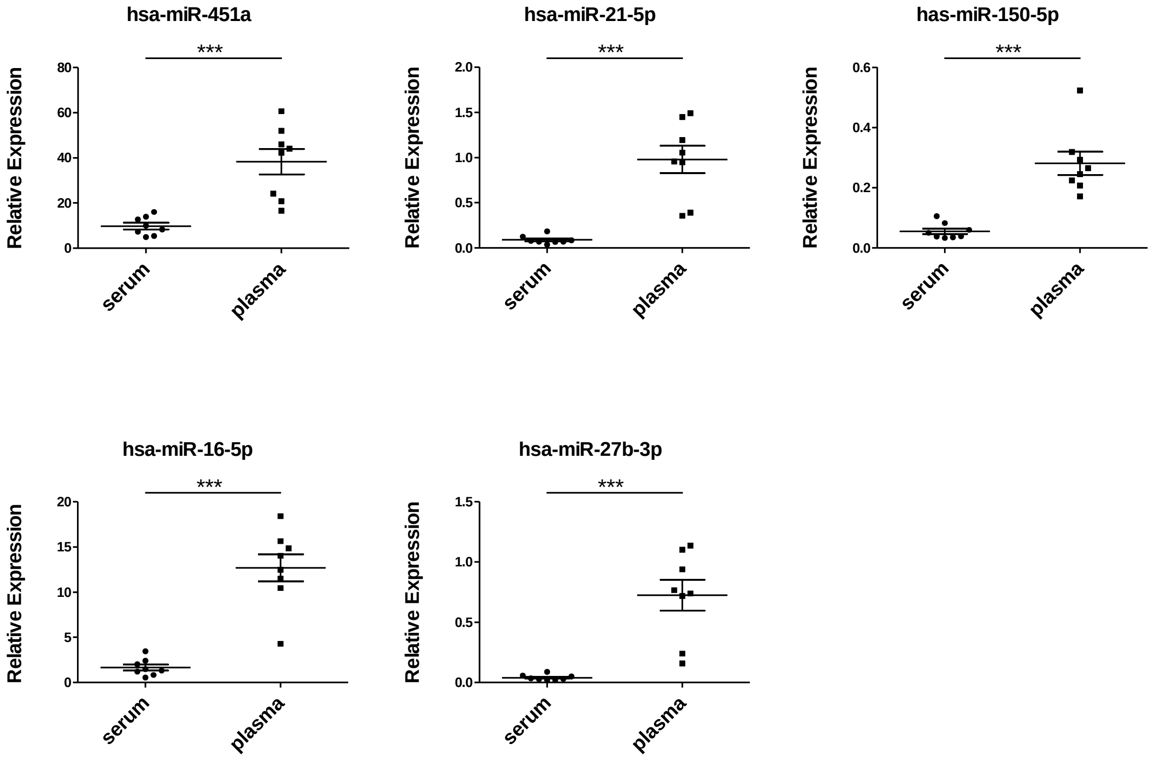

Supplement: Supplementary file 2 — Additional file 2: Figure S1. Expression levels of has-miR-451a, has-miR-150-5p, has-miR-27b-3p and has-miR-92a-3p using purified RNA as template with the S-Poly(T) Plus method . Each volume of plasma and corresponding serum were from a same healthy donor. miRNA levels were normalized to spiked-in cel-miR-54-5p. Data are shown as means ± SE, ***p < 0.001. [file 12967_2019_2061_MOESM2_ESM.pdf]
