# Supplementary material for: Direct S-Poly(T) Plus assay in quantification of microRNAs without RNA extraction and its implications in colorectal cancer biomarker studies
Source: J Transl Med. 2019 Sep 23;17:316. doi: 10.1186/s12967-019-2061-6 (PMC6757382; doi:10.1186/s12967-019-2061-6)

Additional file 3: Figure S2. Comparison of four kinds of DNA polymerase in the Direct S-Poly(T) Plus assay. Plasma was used as template. Alpha taq, Superm taq and Omni taq were purchased from VitaNavi company (VitaNavi, St. Louis USA) and HSSM taq were purchased from Geneup company (Geneup, Shenzhen, China).

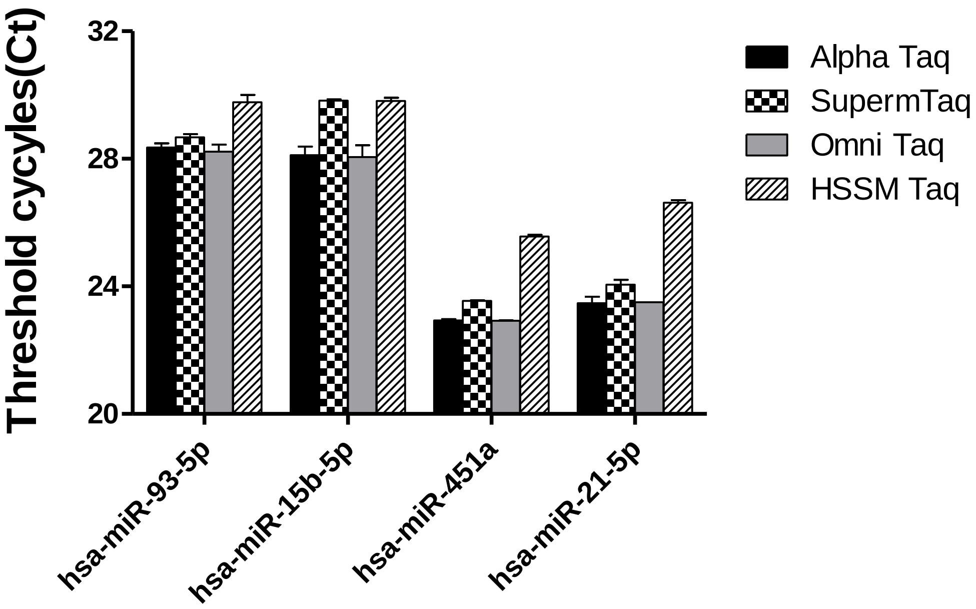

Supplement: Supplementary file 3 — Additional file 3: Figure S2. Comparison of four kinds of DNA polymerase in the Direct S-Poly(T) Plus assay. Plasma was used as template. Alpha taq, Superm taq and Omni taq were purchased from VitaNavi company (VitaNavi, St. Louis USA) and HSSM taq were purchased from Geneup company (Geneup, Shenzhen, China). [file 12967_2019_2061_MOESM3_ESM.pdf]
