# Supplementary material for: Direct S-Poly(T) Plus assay in quantification of microRNAs without RNA extraction and its implications in colorectal cancer biomarker studies
Source: J Transl Med. 2019 Sep 23;17:316. doi: 10.1186/s12967-019-2061-6 (PMC6757382; doi:10.1186/s12967-019-2061-6)

Additional file 4: Figure S3. Expression pattern of 104 miRNAs. Heatmap depicted miRNAs differentially expressed between healthy (NC) and colorectal cancer (CRC) pooled samples. miRNAs were detected with Direct S-Poly(T) Plus method.

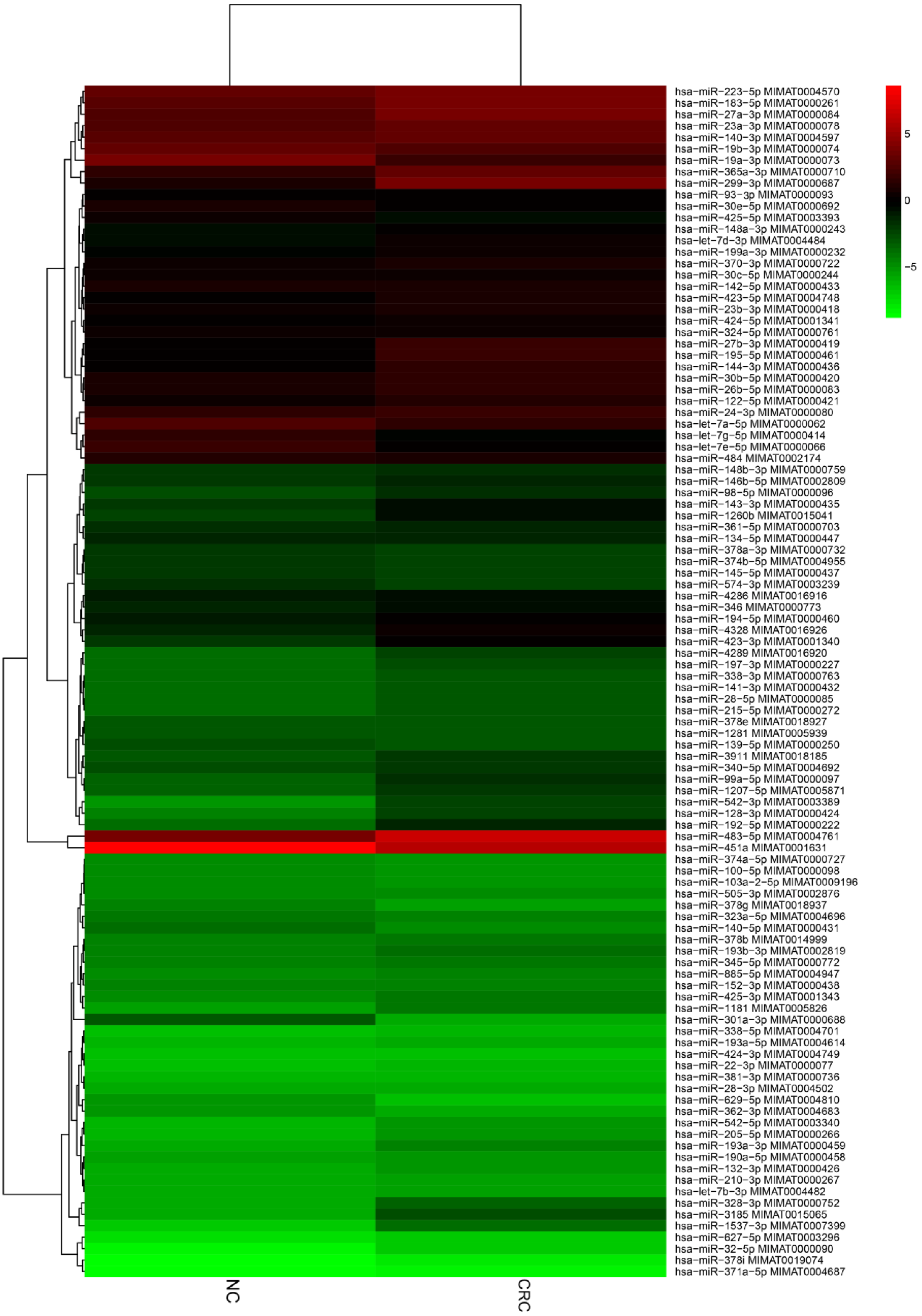

Supplement: Supplementary file 4 — Additional file 4: Figure S3. Expression pattern of 104 miRNAs. Heatmap depicted miRNAs differentially expressed between healthy (NC) and colorectal cancer (CRC) pooled samples. miRNAs were detected with Direct S-Poly(T) Plus method. [file 12967_2019_2061_MOESM4_ESM.pdf]
