# Supplementary material for: Direct S-Poly(T) Plus assay in quantification of microRNAs without RNA extraction and its implications in colorectal cancer biomarker studies
Source: J Transl Med. 2019 Sep 23;17:316. doi: 10.1186/s12967-019-2061-6 (PMC6757382; doi:10.1186/s12967-019-2061-6)

Additional file 5: Figure S4. Differentially expressed miRNAs in 38 plasma samples of colorectal cancer patients and 38 healthy individuals with the Direct S-Poly(T) Plus. Data are shown as means ± SE, *p<0.05, ***p<0.001, ns, not significant.

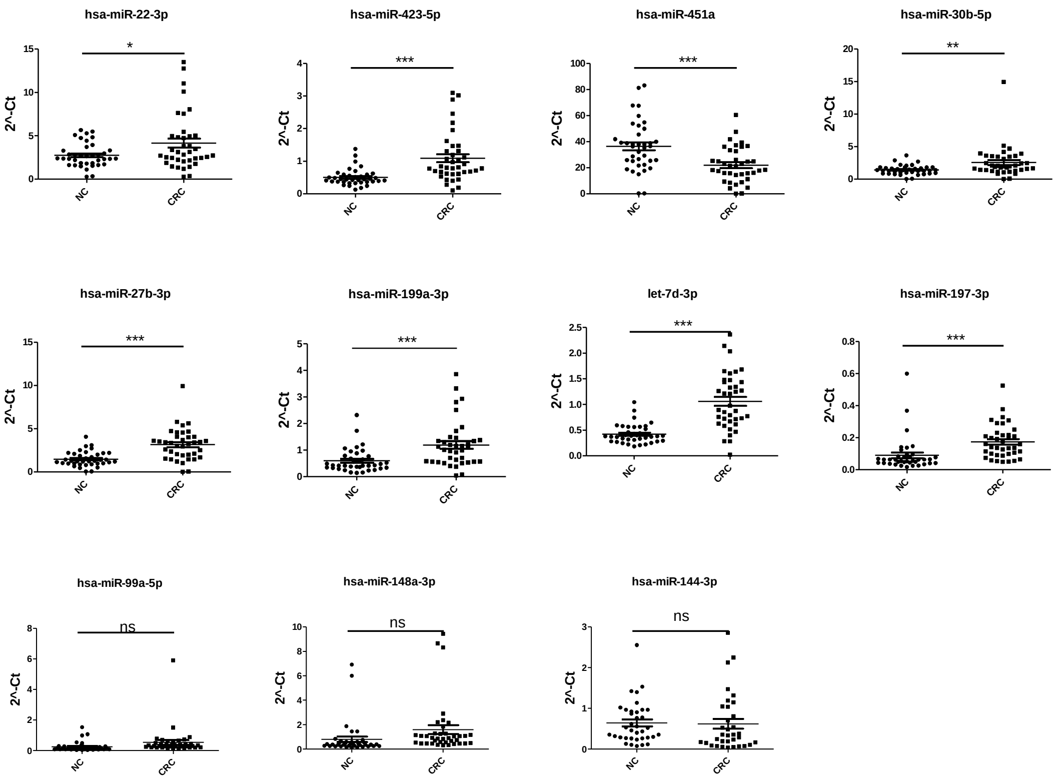

Supplement: Supplementary file 5 — Additional file 5: Figure S4. Differentially expressed miRNAs in 38 plasma samples of colorectal cancer patients and 38 healthy individuals with the Direct S-Poly(T) Plus. Data are shown as means ± SE, *p < 0.05, ***p < 0.001, ns, not significant. [file 12967_2019_2061_MOESM5_ESM.pdf]
