# Supplementary material for: Direct S-Poly(T) Plus assay in quantification of microRNAs without RNA extraction and its implications in colorectal cancer biomarker studies
Source: J Transl Med. 2019 Sep 23;17:316. doi: 10.1186/s12967-019-2061-6 (PMC6757382; doi:10.1186/s12967-019-2061-6)

**Additional file 6: Figure S5.** The sequencing information of 7 potential miRNA biomarkers and reference miRNA. A. Alignment of mature miRNAs and sequencing results; B. Sanger sequencing peak and quality; C. Schematic diagram of cloning RT-qPCR products to plasmid vectors.

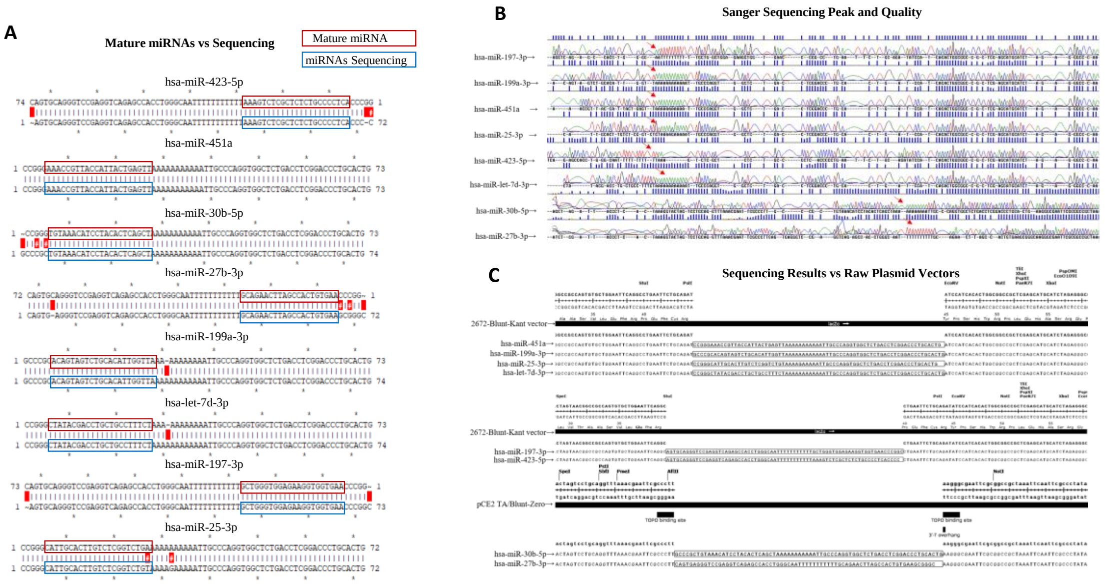

Supplement: Supplementary file 6 — Additional file 6: Figure S5. The sequencing information of 7 potential miRNA biomarkers and reference miRNA. A. Alignment of mature miRNAs and sequencing results; B. Sanger sequencing peak and quality; C. Schematic diagram of cloning RT-qPCR products to plasmid vectors. [file 12967_2019_2061_MOESM6_ESM.pdf]
