# Supplementary material for: Direct S-Poly(T) Plus assay in quantification of microRNAs without RNA extraction and its implications in colorectal cancer biomarker studies
Source: J Transl Med. 2019 Sep 23;17:316. doi: 10.1186/s12967-019-2061-6 (PMC6757382; doi:10.1186/s12967-019-2061-6)

Additional file 7: Figure S6. Differentially expressed miRNAs in 36 serum samples of colorectal cancer patients and 36 controls with the Direct S-Poly(T) Plus. Serum samples were collected from Cancer Center of Guangzhou Medical University (Guangzhou). Data are shown as means ± SE, ***p*<0.01, ****p*<0.001, ns, not significant.

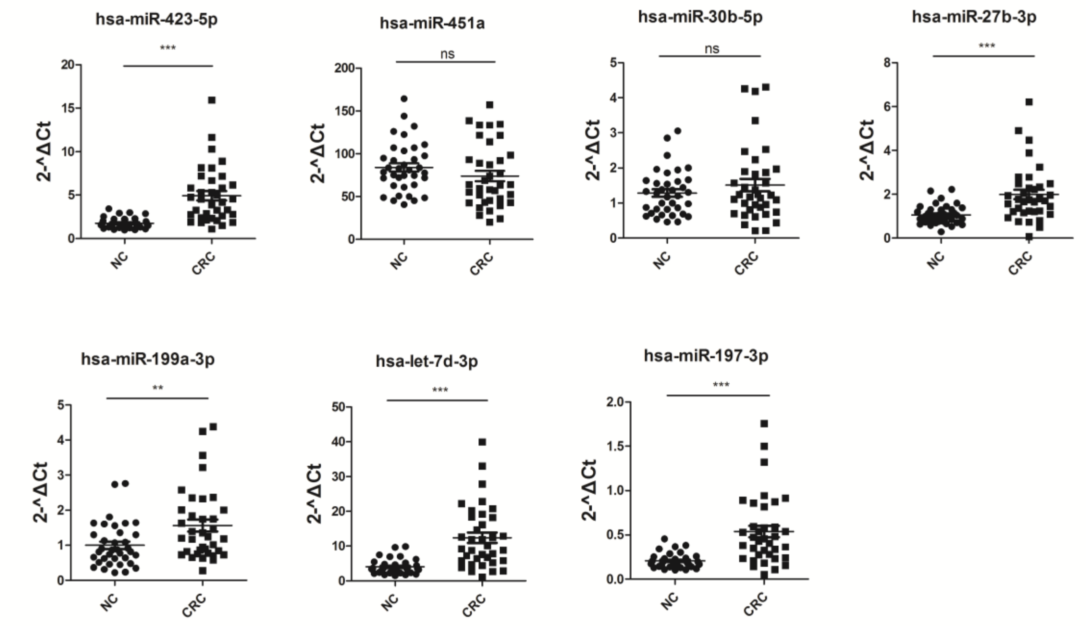

Supplement: Supplementary file 7 — Additional file 7: Figure S6. Differentially expressed miRNAs in 36 serum samples of colorectal cancer patients and 36 controls with the Direct S-Poly(T) Plus. Serum samples were collected from Cancer Center of Guangzhou Medical University (Guangzhou). Data are shown as means ± SE, **p < 0.01, ***p < 0.001, ns, not significant. [file 12967_2019_2061_MOESM7_ESM.pdf]
